# A pipeline for automatic bipolar disorder analysis through anonymization and LLM-as-a-judge

**DOI:** 10.1192/j.eurpsy.2026.10414

**Published:** 2026-07-31

**Authors:** A. Tremopoulos, M. De Prisco, A. Mas-Musons, C. Valenzuela-Pascual, M. Korniyenko, V. Oliva, G. Fico, J. Raduà, E. Vieta, G. Anmella, D. Hidalgo-Mazzei, C. Escolano

**Affiliations:** 1 Polytechnic University of Catalonia (UPC); 2 Hospital Clínic of Barcelona, Bipolar and Depressive Disorders Unit, IDIBAPS, CIBERSAM, University of Barcelona, Barcelona, Spain; 3 Imaging of Mood- and Anxiety-Related Disorders (IMARD) group, IDIBAPS, Barcelona, Spain., Barcelona, Spain

## Abstract

**Introduction:**

Bipolar Disorder (BD) is a psychiatric condition characterized by marked shifts in mood, energy, and thought processes, manifested in episodes of mania, depression, and euthymia. Despite advances in digital psychiatry, few studies have systematically assessed the potential of Large Language Models (LLMs) to analyze real clinical interviews in BD, particularly in multiple languages.

**Objectives:**

This work presents a pipeline designed to (1) anonymize real psychiatric interviews while preserving clinical information, and (2) evaluate the capabilities of an open-source LLM, Qwen2-Audio, to classify affective states and their severity in BD.

**Methods:**

A dataset of 41 audio recordings in Spanish and Catalan was used, covering different illness phases. A complete anonymization pipeline was developed to remove sensitive information from transcripts while extracting audio embeddings from the LLM’s internal representation. These embeddings were explored in latent space to assess separability of affective states. A structured chain-of-thought (CoT) prompting strategy was then implemented, asking the model symptom-based clinical questions to emulate psychiatric reasoning. Multiple input modalities (audio, text, audio+text, and full transcripts) and prompt strategies were compared across sessions.

**Results:**

The anonymization pipeline effectively protected patient confidentiality while enabling the use of real clinical data. Audio embeddings revealed meaningful differences between affective states within the same patient. Prompt design strongly influenced performance, with CoT prompting reducing hallucinations and improving consistency. Audio input achieved the best performance for depression (recall = 88.41%), full-text input was most effective for mania detection (F1 = 59%), while euthymia remained challenging (max F1 = 46.2%). Severity predictions were more accurate for higher severity levels (e.g., moderate depression F1 ≈ 70%, severe mania F1 = 53.2%).

**Conclusions:**

This work demonstrates the feasibility of combining anonymization pipelines, real psychiatric data, and open-source multimodal LLMs for BD analysis. By jointly evaluating depression, mania, and euthymia, and by applying structured CoT reasoning, the study fills an important gap in the literature and provides a reproducible, privacy-preserving framework for future research in digital psychiatry.

**Disclosure of Interest:**

None Declared

